# Preimplantation Exposure to Bisphenol A and Triclosan May Lead to Implantation Failure in Humans

**DOI:** 10.1155/2015/184845

**Published:** 2015-08-19

**Authors:** Mu Yuan, Ming-Zhu Bai, Xu-Feng Huang, Yue Zhang, Jing Liu, Min-Hao Hu, Wei-Qian Zheng, Fan Jin

**Affiliations:** ^1^Department of Reproductive Endocrinology, Women's Hospital, School of Medicine, Zhejiang University, 1 Xueshi Road, Hangzhou 310006, China; ^2^Department of Obstetrics and Gynecology, Shanghai First People's Hospital, Shanghai Jiaotong University, Shanghai 201600, China; ^3^School of Medicine, University of Wollongong and Illawarra Health and Medical Research Institute, Wollongong, NSW 2522, Australia

## Abstract

Endocrine disrupting chemicals (EDCs) are chemicals that have the capacity to interfere with normal endocrine systems. Two EDCs, bisphenol A (BPA) and triclosan (TCS), are mass-produced and widespread. They both have estrogenic properties and similar chemical structures and pharmacokinetic features and have been detected in human fluids and tissues. Clinical evidence has suggested a positive association between BPA exposure and implantation failure in IVF patients. Studies in mouse models have suggested that preimplantation exposure to BPA and TCS can lead to implantation failure. This paper reviews the relationship between preimplantation exposure to BPA and TCS and implantation failure and discusses the remaining problems and possible solutions.

## 1. Introduction

During the last few decades, the incidence of human infertility has significantly increased in many countries, such as the United States and China [1–5]. The rate of this increase is too rapid to be explained by genetic mutations. More than 10% of infertile couples suffer from infertility of an unexplained nature [6]. The women in these couples have normal ovulatory cycles and hormonal profiles and no organ pathologies. Their partners show no evidence of semen quality problems. In the meantime, the production of many artificial chemicals such as plastics has been increased [7].

**Figure 1:**
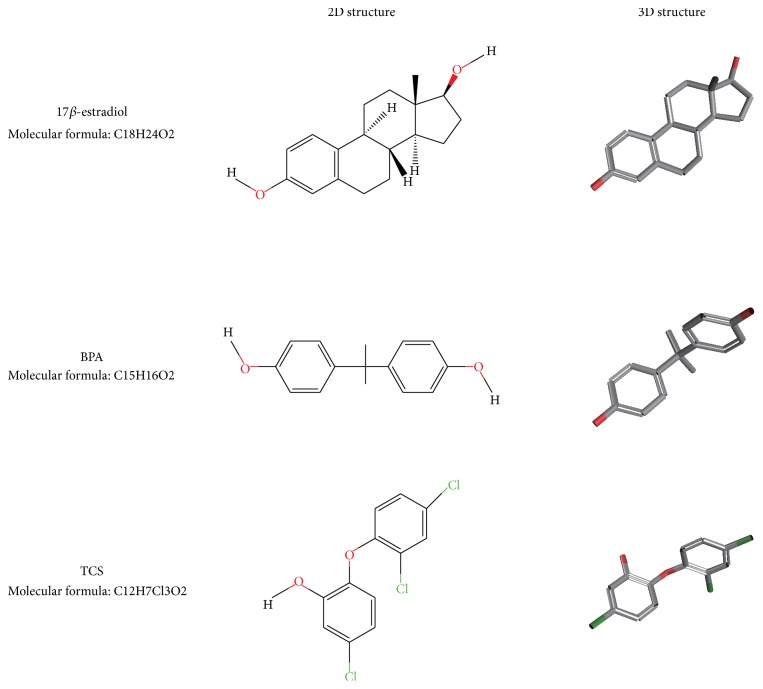
Chemical structures of BPA, TCS, and 17*β*-estradiol.

Some chemicals which have been widely used for decades have recently been found to have the ability to disrupt endocrine function in humans. They are called endocrine disrupting chemicals (EDCs). Bisphenol A (BPA) and triclosan (TCS) are EDCs with similar chemical structures to 17*β*-estradiol [8] (Figure 1). They have recently been noticed due to their ubiquitous presence in the environment and in human fluids and tissues [9–23]. BPA is the monomer used in the production of polycarbonate plastics and some epoxy resins. It is one of the most-produced chemicals worldwide, with over six billion pounds produced each year [21]. TCS is an antimicrobial additive used in many personal care and household products.

Many studies have suggested that BPA exposure is associated with female infertility [15–17]. However, the association between TCS exposure and female infertility remains unknown. Mice have been used as an animal model to study the association between the exposure to these two chemicals and infertility [24–28].

Subtle changes in estrogen levels can lead to implantation failure in humans and mice [29, 30]. BPA and TCS have estrogenic activity in vitro and in vivo [21, 31]. BPA binds to both ER*α* and ER*β* [32–35]. Both BPA and TCS have many biological effects mediated via estrogen receptors [36–39]. Thus, BPA and TCS may cause implantation failure due to their ability to mimic estrogen in humans [40–42]. In human beings, from oocyte maturation to implantation, the biological features of the oocyte and the embryo change dramatically. The levels of sex hormones, such as estrogen, progesterone, and androgen, and their receptors also change dramatically. Thus, the sensitivity of the female reproductive system to BPA and TCS may vary depending on the time of exposure. It has been reported that, in mice, preimplantation exposure to the same amount of BPA or TCS on gestational day 2/3 is more potent to induce embryo implantation failure than exposure on gestational day 0/1 [27, 28]. Thus, in mice, gestational day 2-3 may be a sensitive window for BPA and TCS. Exposure to these two endocrine disruptors during a sensitive window might lead to implantation failure. However, in human beings, the sensitive window for these EDCs still needs further investigation.

## 2. Exposure and Detection of BPA and TCS in Humans

### 2.1. The Route and Amount of Exposure to BPA and TCS in Humans

There is a trend of increased exposure to endocrine disrupting chemicals, including BPA and TCS. In 2008, it was reported that daily exposure of BPA to humans is below 0.1 *μ*g/kg/day for the majority of the population [43]. In 2011, Taylor et al. suggested that total daily human exposure of BPA is via multiple routes and is much higher than previously assumed based on animal studies and pharmacokinetic features of BPA in human and animal [44]. Recently, Lassen et al. [45] reported that the median daily intake of BPA among 33 Danish men is approximately 27 ng/kg/day.

Although BPA and TCS can be absorbed orally, dermally, and by inhalation [46], the majority of absorption occurs via ingestion. It is estimated that 90–99% of BPA exposure in adults and children is from food [47–49]. Allmyr et al. [50] suggested that oral care products are probably the most important means of exposure to TCS in adults because brushing teeth with TCS-containing toothpaste has been shown to result in a large and rapid uptake of TCS. However, the percentage of TCS ingested orally in relation to total TCS absorption is not known.

Rodricks et al. [51] estimated the amount of human daily exposure to TCS from two approaches. One approach is based on the estimation of the combination of daily intake products, and the other is based on biomonitoring data from human volunteers. The total intake of TCS per day from the consumer products evaluated were 0.047, 0.065, and 0.073 mg/kg/day for men, women, and children, respectively [51]. The daily TCS intake estimates based on the 50th percentile urinary concentrations of TCS reported in the NHANES (National Health and Nutrition Examination Survey) 2003-2004 were approximately 0.0002, 0.0002, and 0.0001 mg/kg/day for men, women, and children, respectively [51]. The estimates based on the 95th percentile urinary concentrations of TCS were approximately 0.009, 0.007, and 0.004 mg/kg/day [51]. The estimated exposure level of TCS based on biomonitoring data is much higher than the product-based TCS intake estimates and suggests that actual TCS intakes are lower than the product-based estimates.

People living in different regions or having different living habits are probably exposed to different amounts of these two EDCs. And the human exposure level of BPA and TCS remains unclear and needs further investigation.

### 2.2. Distribution and Amount of TCS and BPA in Human Tissue

BPA and TCS have been detected in the blood, breast milk, urine, adipose tissue, liver, and brain of most human volunteers [9–21]. BPA has also been detected in human amniotic fluid and follicular fluid [22, 23]. The distribution and amount of BPA and TCS in the human body are listed in Table 1. It is interesting that BPA has an ~5-fold higher concentration at 15–18-week gestation, which must be considered in evaluating the potential for human exposure to BPA [22]. The concentrations of BPA in serum, breast milk, follicular fluid, amniotic fluid (full term), and urine are very close, suggesting that BPA is distributed evenly in human fluids. The distribution and amount of BPA and samples suggest a profile of ubiquitous presentation in human body.

## 3. Pharmacokinetics of BPA and TCS

### 3.1. BPA

Völkel et al. [52] administered 5 mg radioactive BPA/person (54–90 ug/kg body weight) and reported that this BPA was completely eliminated from the body within 24 h. Maximum plasma concentrations were reached 80 min after dosing and rapidly declined for the next 6 h. BPA is only detected in its glucuronidated form and not as free BPA. This study indicates that BPA was absorbed from the gastrointestinal tract quickly, conjugated with glucuronic acid in the liver, and BPA-glucuronide was rapidly filtered from the blood by the kidneys and excreted in the urine [52]. Acute studies in both mice and humans indicate rapid metabolism and clearance of BPA [44, 52, 53].

### 3.2. TCS

The absorption of TCS following oral administration in both humans and mice is rapid and efficient [51]. Maximum plasma concentrations were achieved 1 to 3 h following administration in humans and 1 to 4 h in mice, respectively [51, 54]. In humans, TCS does not accumulate in the blood [55]. Nearly all of the TCS absorbed is metabolized to sulfate and glucuronide conjugates in both humans and mice [51, 56, 57]. In humans, about 80–85% of the administered dose in volunteers is excreted in the urine (71–80%) or feces (5–7%) in the form of conjugated metabolite, and the elimination half-life of TCS is estimated to be approximately 10 to 20 h [51, 54]. Most of the absorbed TCS can be excreted from the human body less than 24 h after exposure [54].

In short, the pharmacokinetic features of TCS are very similar to BPA in both humans and mice. In both humans and mice, TCS can be rapidly and nearly completely absorbed, metabolized to glucuronide and sulfate conjugates, and excreted.

## 4. Preimplantation Exposure to BPA and TCS Can Cause Implantation Failure

Accumulating evidence suggests that there is an association between women's exposure to BPA and female infertility. Moreover, animal model studies have suggested that exposure to BPA, TCS, or both BPA and TCS during the preimplantation period could lead to implantation failure in mice.

### 4.1. Clinical Studies

In 2005, Sugiura-Ogasawara et al. [17] reported that BPA is associated with recurrent miscarriage in humans. Recently, Ehrlich et al. [15] reported a significant linear dose-response association between increased urinary BPA concentrations and a decreased number of oocytes (overall and mature), a decreased number of normally fertilized oocytes, and decreased peak serum estradiol levels. The mean number of oocytes and normally fertilized oocytes decreased by 24 and 27%, respectively, for the highest versus the lowest quartile of urinary BPA (trend test *P* < 0.001 and 0.002, resp.). Women with urinary BPA above the lowest quartile had decreased blastocyst formation (trend test *P* = 0.08). Ehrlich et al. [16] also claimed a positive linear dose-response association between BPA urinary concentrations and implantation failure.

The correlation between the concentration of TCS in pregnant women and female infertility remains unclear due to a lack of investigation. However, the similarity of the distribution, chemical structures, and estrogenic activity of BPA and TCS suggests the possible involvement of TCS in implantation failure.

### 4.2. Animal Studies

Implantation failure could be caused by the embryo itself, inadequate uterine receptivity, or defects in communication between the embryo and the endometrium. It is generally assumed that the embryo itself is probably only responsible for one-third of IVF failures, and the other two-thirds of implantation failures occur due to impaired uterine receptivity or defects in embryo-endometrium communication [58–60]. Animal models have been used to investigate the effect of preimplantation exposure of BPA and TCS on implantation.

Crawford and deCatanzaro [28] studied the impact of preimplantation exposure to BPA, TCS, and BPA and TCS on implantation rates in mice. They found that exposure to TCS on the level of 523/785 mg/kg/day on gestational days (GD) 1–3 could significantly reduce implantation rates by 30%/40% [28]. They also found that although doses of 4 mg BPA (122 mg/kg) and 9 mg triclosan (262 mg/kg) on GD 1–3 were individually ineffective, in combination they reduced the number of implantation sites and also increased gestation length [28]. Xiao et al. [24] showed that preimplantation exposure to 100 mg/kg/day BPA on gestational days 0.5–3.5 can reduce implantation rates to zero by affecting uterine receptivity, embryo transport, and preimplantation embryo development in mice. Berger et al. [25] reported that preimplantation exposure to 200/300 mg/kg/day BPA on gestational days 1–4 can reduce implantation sites by 70%/100%.

Takai et al. [61] showed that, at 100 uM, BPA could inhibit the development of preimplantation embryos in vitro. At lower, more environmentally relevant concentrations (1 nM and 3 nM), BPA has stimulatory effects on embryo development in mice.

### 4.3. Possible Mechanisms

Uterine receptivity and embryo development are both critical for successful implantation. Coordinated actions of progesterone and estrogen play a critical role in creating a receptive uterine environment, embryo development, and embryo migration through the oviduct [62, 63]. Estrogen and progesterone actions are critical in the regulation of uterine cell proliferation, establishing a window of receptivity for blastocyst implantation [64, 65]. In mice, this window is very narrow and sensitive to changes in steroid levels [29, 62]. Small increases in estradiol levels can alter uterine PR (progesterone receptor) and gene expression, causing the uterus to enter a refractory state and thereby decreasing the probability of successful implantation [29]. Kim et al. [66] claimed that, through nuclear ER-dependent ERK1/2 phosphorylation, both E2 and BPA can rapidly and transiently induce Egr1 which may be important for embryo implantation and decidualization in mouse uterus. Recently, Mannelli et al. [67] reported that BPA perturbed the expression of ER*α*, ER*β*, PRA, PRB, and hCG/LH-R, reduced the mRNA transcription of dPRL, and stimulated secretion of MIF in human endometrial stromal cells in vitro.

Several studies have shown that BPA can impair the development of mouse embryos and that this effect can be counteracted by Tamoxifen (an estrogen receptor modulator) [24, 61, 68]. Thus, the impairment of BPA on the embryo is probably mediated by the estrogen receptor.

BPA, TCS, and 17*β*-estradiol have similar chemical structures and they are all fat-soluble chemicals. BPA and TCS both have estrogenic activity, share similar pharmacokinetic features, and can be detected in human fluids and tissues. Evidence from clinical studies and animal models supports the assumption that preimplantation exposure to BPA and TCS could lead to implantation failure in humans.

In addition, preimplantation exposure to BPA can nonmonotonically change the expression of the ER*α* (estrogen receptor *α*) and PR (progesterone receptor) in the uterus of mice [25]. Thus it seems that BPA might interfere with the coordinated actions of progesterone and estrogen and impair the receptivity of the uterus and embryo migration. Xiao et al. [24] reported that preimplantation exposure to BPA affects embryo transport, preimplantation embryo development, and uterine receptivity in mice.

BPA also can increase the luminal area and luminal cell height of the mouse uterus on gestational day 6 following subcutaneous injections of BPA on days 1–4 of gestation [25]. These morphological changes in the uterus could have implications for the success of blastocyst implantation.

Varayoud et al. [69] reported that neonatal exposure to BPA alters rat uterine HOXa10 and its downstream gene expression and reduces the number of implantation sites compared to the control group. Bromer et al. [70] claimed that BPA exposure in utero on gestational days 9–16 (after implantation and in the middle of the pregnancy) can upregulate the expression of HOXa10 in the uterus of female offspring in mice. However, whether preimplantation exposure to BPA affects the expression of HOXa10 and its downstream genes has not been investigated.

Some studies have suggested that exposure to BPA could affect the meiotic maturation of oocytes in humans and mice [71–76].

**Figure 2:**
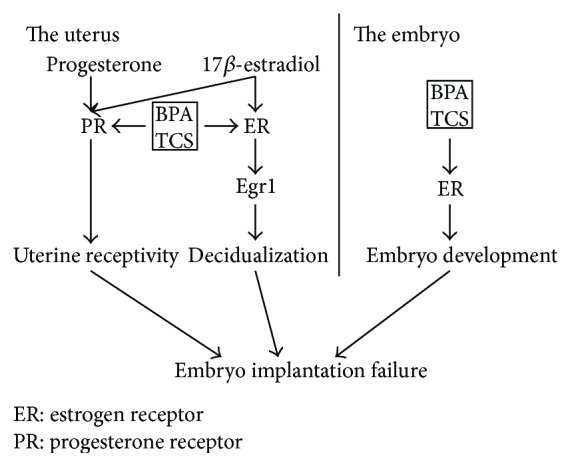
Possible mechanism by which BPA and TCS may affect embryo implantation.

Crawford and deCatanzaro [28] suggested that preimplantation exposure to TCS can also cause implantation failure in mice and that TCS can act in conjunction with BPA. However, the mechanism of this implantation failure in humans and mice is still unknown. The possible mechanism by which the two chemicals may affect embryo implantation is illustrated in Figure 2.

## 5. Summary and Suggestions for Further Studies

In the clinic, fertilization can only be confirmed afterwards. There is no way to detect embryo implantation failure in the clinic except for patients undergoing in vitro fertilization. The most common way to diagnose pregnancy is by testing human chorionic gonadotrophin (HCG) in urine samples. However, HCG is secreted by the syncytiotrophoblast and is detectable in maternal blood two days after the implantation of the embryo [77]. Thus, it is possible that many women did not know that they had a fertilized embryo which failed to implant into their endometrium because no HCG was secreted. Even if there was vaginal bleeding and they went to see a gynecologist, it will only be seen as ovulation bleeding which is very common in the clinic. Most of the time, when a woman wants to know if she is pregnant she will do a urine pregnancy test. However, a detectable level of HCG in the urine requires the embryo to survive for at least a week after implantation. Since BPA and TCS can be absorbed and excreted quickly and do not accumulate in the human body [44, 51–53], a change in habits like starting or stopping using TCS-containing toothpaste or using plastic food containers can cause a fluctuation in the levels of these two chemicals in the human body. This means that if the exposure ceased during the sensitive time frame—for example, the woman has used up her TCS-containing toothpaste and bought some new TCS-free toothpaste or lost her plastic bottles which leak BPA—she could have a relatively low level of TCS and BPA in her body during this time and become pregnant. In the clinic, a woman cannot be defined as infertile unless she has attempted unprotected coitus for at least one year without becoming pregnant. This means that perhaps BPA and TCS have caused more miscarriages than we have realized.

However, the most sensitive time for BPA and TCS to influence implantation remains unknown. Although the preimplantation period might be a sensitive time frame for BPA and TCS exposure, it might not be the most sensitive and important one.

The procedure of in vitro fertilization and embryo transfer (IVF/ET) has provided some possible ways to identify the most dangerous time frame for TCS and BPA exposure in humans. Only in those patients would we know the exact time of fertilization, the condition of the oocyte prefertilization, and the embryo's preimplantation.

Recently, Ehrlich et al. [15, 16] studied the association between BPA exposure levels and the clinical outcomes of IVF patients as mentioned above. However, their study did not measure the exposure level of TCS in those patients or the chromosomal condition of the oocytes before fertilization using techniques such as biopsy of their first polar body or the discarded GV (germinal vesicle) oocytes (i.e., immature oocytes). Oocytes with meiotic abnormalities can look normal and even become fertilized, but they lead to a low fertilization rate or a low implantation rate if they are fertilized [78, 79].

Many studies have investigated the association between BPA exposure and clinical outcomes in IVF patients [14–16, 18]. However, the menstrual cycles of IVF patients are not natural but are altered using hormones to obtain more ova and the optimal endometrium state during embryo transfer. Since it is likely that BPA and TCS act through estrogen receptors, it is best to study their effect under natural conditions where the status of estrogen receptors and the amount of estrogen in vivo are less affected by exogenous hormones. For example, one could conduct the study in IVF patients undergoing natural cycles. Since only patients with stable menstrual cycles would be included in this procedure, the samples may be more monotonous. Also, most of the animal studies have used the subcutaneous route for exposure [25, 27, 28, 69]. However, human exposure to BPA and TCS is mainly orally. So either gavage or food and drink might be a better exposure procedure for animal models.

It is not easy to maintain low TCS and BPA exposure levels due to their widespread existence. And recently it has been reported that BPA-free plastic products also release other chemicals with estrogenic activity [80]. Fortunately, the pharmacokinetic features of these two chemicals suggest that the fertility rate of women could probably be raised just by minimizing contact with BPA- and TCS-containing products during sensitive time frames such as the preimplantation period. However, the other part of reproduction, including the period of the second meiosis of the oocyte, cannot be excluded. If we could determine the most sensitive time frame for these two chemicals, we might not need to find a counteractive drug.

## Figures and Tables

**Table 1:** The distribution and amount of BPA and TCS in human tissue.

Chemical	Tissue type	Concentration	References
BPA	Serum (adult)	~1-2 ng/mL	[22, 81]
	Serum (fetal)	~1-2 ng/mL	[22]
	Breast milk	0.61 ± 0.20 ng/mL	[82]
	Colostrum	3.41 ± 0.13 ng/mL	[83]
	Follicular fluid	~1-2 ng/mL	[22]
	Amniotic fluid (full term)	~1-2 ng/mL	[22]
	Amniotic fluid (15–18-week gestation)	8.3 ± 8.7 ng/mL	[22]
	Urine	2.75–3.3 *μ*g/g creatinine (~3.00 ng/mL)	[84, 85]
	Brain	0.91 ng/g	[10]
	Adipose tissue	3.78–5.83 ng/g	[10, 86]
	Liver	1.48 ng/g	[10]

TCS	Serum	4.1–19 ng/g	[50]
	Breast milk	1.3 ± 2.7 ng/g fresh weight	[87]
	Urine	3.55 *μ*g/g creatinine (3.77 ng/mL)	[84]
	Adipose tissue	0.61 ng/g	[10]
	Liver	3.14 ng/g	[10]
